# Oligomerization and Phosphorylation Dependent Regulation of ArgBP2 Adaptive Capabilities and Associated Functions

**DOI:** 10.1371/journal.pone.0087130

**Published:** 2014-01-27

**Authors:** Julie Roignot, Thomas Bonacci, Eric Ghigo, Juan L. Iovanna, Philippe Soubeyran

**Affiliations:** 1 Centre de Recherche en Carcérologie de Marseille (CRCM), INSERM UMR 1068, CNRS UMR 7258, Aix-Marseille University and Institut Paoli-Calmettes, Marseille, France; 2 URMITE-IRD198, CNRS UMR7278, INSERM U1095, Aix-Marseille Univ, Marseille, France; Aix-Marseille University, France

## Abstract

ArgBP2 (Arg-Binding Protein 2/SORBS2) is an adaptor protein involved in cytoskeleton associated signal transduction, thereby regulating cell migration and adhesion. These features are associated with its antitumoral role in pancreatic cancer cells. Tyrosine phosphorylation of ArgBP2, mediated by c-Abl kinase and counterbalanced by PTP-PEST phosphatase, regulates many of its interactions. However, the exact mechanisms of action and of regulation of ArgBP2 remain largely unknown. We found that ArgBP2 has the capacity to form oligomers which are destabilized by tyrosine phosphorylation. We could show that ArgBP2 oligomerization involves the binding of one of its SH3 domains to a specific proline rich cluster. ArgBP2 self-association increases its binding to some of its molecular partners and decreased its affinity for others. Hence, the phosphorylation/oligomerization state of ArgBP2 directly regulates its functions by modulating its adaptive capabilities. Importantly, using a human pancreatic cancer cell model (MiaPaCa-2 cells), we could validate that this property of ArgBP2 is critical for its cytoskeleton associated functions. In conclusions, we describe a new mechanism of regulation of ArgBP2 where tyrosine phosphorylation of the protein interfere with a SH3 mediated self-interaction, thereby controlling its panel of interacting partners and related functions.

## Introduction

ArgBP2 (SORBS2), together with CAP/Ponsin and Vinexin, belongs to the SoHo (Sorbin Homology) family of adaptor proteins. These proteins, which contain the unique Sorbin Homology domain in their N-terminal half and three SH3 domains in their C-terminus, are well known to be involved in regulating cytoskeleton associated cell signaling events such as cell adhesion, cell migration, or vesicle trafficking [1], [2]. CAP was shown to play a critical role in insulin signaling [3]. It enables the translocation of the ubiquitin ligase Cbl to lipid rafts microdomains permitting the activation of the small GTPase TC10. This activation is essential to provoke the export of GLUT4 receptors from intracytoplasmic citterns to the plasma membrane [3], [4], [5]. Vinexin has been implicated in the regulation of the spreading and migration of different cell types, in anchorage independent growth and in various cell signaling events [6], [7], [8], [9], [10]. ArgBP2 has been involved in regulatory processes of cytoskeleton organization and dynamic [11], [12], [13], synapses physiology [14] and neurites growth [15], regulation of cytoplasmic kinases [16], [17], tight junctions organization [18], and stability of vascular lumens [19]. We previously observed that these functions of ArgBP2 are important for normal behavior of pancreatic cells, and loss of ArgBP2 in pancreatic cancer cells is associated with increased aggressiveness [20], [21]. This antitumoral function of ArgBP2 depends on its ability to control the multiple interactions of its numerous molecular partners, especially those involved in actin dynamics regulation such as WAVEs (wiskott-Aldrich syndrome protein containing Verprolin homology domain) [22], c-Abl (Abelson kinase) [23], CIP4 (Cdc42 interacting protein 4) [24], and PTP-PEST (Protein tyrosine phosphatase with PEST domain) [25], and has been observed in other type of cancers [26], [27], [28].

Part of ArgBP2's functions is known to be regulated by its phosphorylation. In fact, ArgBP2 has been identified as a protein interacting with c-Arg and c-Abl kinases, and was shown to be strongly phosphorylated by both kinases [13]. Tyrosine phosphorylation of ArgBP2, which is inhibited by the action of PTP-PEST phosphatase [21], as well as its serine phosphorylation by PAK1 [17], have been shown to control functions of ArgBP2 by enhancing its binding to a fraction of its molecular partners and inhibiting its association with others. A negative regulation of ArgBP2 and of some of its associated proteins, such as c-Abl, can be achieved by another ArgBP2 associated protein, the ubiquitin ligase c-Cbl, which mediates their polyubiquitination followed by proteasomal degradation [16]. Importantly, tyrosine phosphorylation of ArgBP2 increases this c-Cbl mediated negative regulation of ArgBP2 and of c-Abl.

Many interacting partners have been identified for ArgBP2, Vinexin, and CAP, and some of them are shared by two or all the three members of the SoHo protein family [1], [2]. Despite the many pathways these three proteins have been involved in, their exact mechanisms of action and their modes of regulation still need to be clarified. The demonstration that ArgBP2 is able to self-associate in a phosphorylation regulated manner and that this oligomerization plays a major regulatory role regarding its functions provide new clues which highly contribute to the enlightening of these processes.

## Materials and Methods

### Cell culture and transfection

MiaPaCa2, BxPC3, NIH-3T3 and HEK-293T cell lines were obtained from ATCC and maintained according to ATCC's recommendations. MiaPaCa2 cells stably expressing GFP, ArgBP2 WT or ArgBP2 P1 mutant were generated by lentiviral infection. Expression of each protein has been verified by Western Blot and immunofluorescence. HEK-293T, and NIH-3T3 cells were transiently transfected using lipofectamine 2000 reagent (Invitrogen) following manufacturer's instructions. A total of 1 µg of cDNA per well was used when cells were transfected in 12-well plates, 2 µg per well of 6-well plates, and 6 µg in 10 cm dishes. BxPC3 cells were transected with ArgBP2 siRNA as previously described [21].

### Plasmids and peptides

Myc and Flag-ArgBP2 wild type and 5Y mutant, Flag-ArgBP2 N-terminal and C-terminal, GST-ArgBP2 constructs (N-terminal, C-terminal, SH3A, SH3B and SH3C), c-Abl (WT, KD, SH2, and SH3 mutants), Flag-PTP-PEST, Myc-WAVE1 and Flag-CIP4 have been previously described [16], [20], [21]. ArgBP2-P1, -P2, -P3, and -TM were generated by PCR from Flag- and Myc-ArgBP2 WT using QuikChange® site-directed mutagenesis technology (Agilent). Proline residues contained in proline motifs (PXXP) of P1, P2 and P3 clusters were changed to alanines. The forward primers were as follow (mutated codons are underlined):

P497,500A (P1 mutant)

5′GCCTGCAAGAGCACCTCCGGCAGCCCAGCCCGGAGAAATCGGAGAAGCTATAGCCAAATAC-3′

P577,P579A (P2 mutant)

5′GGTGCTGAGGACTACCCTGACCCTGCAATAGCCCACAGCTATTCTAGTGATAGG-3′

P595A (P3 mutant)

5′-GCTTGAGCTCAAATAAGGCACAGCGTCCTGTG-3′

Protein fusions corresponding to Luciferase-ArgBP2 WT and P1 were generated by subcloning the ArgBP2 WT and P1 cDNA in frame in the multiple cloning site of pRluc-C1 humanized vector (PerkinElmer). Protein fusions corresponding to EYFP- ArgBP2 WT and P1 were generated by sub-cloning ArgBP2 WT and P1 cDNA in frame in the multiple cloning site of pEYFP-C1 vector (Clontech). Plasmids used for lentiviral infection (pCCL-ArgBP2 WT and pCCL-ArgBP2 P1) were generated by subcloning ArgBP2 WT and ArgBP2 P1 cDNA in the multiple cloning site of the lentiviral vector plasmid pCCL-WPS-PGK (a kind gift from Dr. Cédric Raoul, INSERM, INMED, Marseille). All cloning products were verified by sequencing. The sequence of the P1 peptide was as follow: Ac-AQPARPPPPAQPGE-NH2. It was obtained from Genscript Corporation and was acetylated at the amino terminus and amidated at the carboxyl terminus.

### Antibodies

The following antibodies and serum were used: rabbit polyclonal anti-ArgBP2 serum ([16], ArgBP2-3SH3), mouse monoclonal anti-ArgBP2 ([21], clone C1), mouse monoclonal anti-Myc (9E10), mouse monoclonal anti-Flag M2 and rabbit anti-GST-HRP (A-7340) were from Sigma-Aldrich, rabbit polyclonal anti-WAVE (H-180), mouse monoclonal anti-phosphotyrosine antibody (PY99), and rabbit polyclonal anti-Abl (K12), were from Santa Cruz Biotechnology.

### Immunoprecipitation, GST pull down, Western Blotting, and Far Western Blotting

24 hours after transfection, cells were lysed in lysis buffer [16] containing protease inhibitors cocktail (Sigma-Aldrich) for 10 min at 4°C, and protein concentration determined with Protein Assay (Bio-Rad). For immunoprecipitation experiments, cleared lysates with adjusted protein concentration were incubated with 2 µg of the suitable antibody for 2 H at 4°C. Immune complexes were precipitated with Protein G/A-sepharose beads for an additional hour at 4°C. After extensive washing in cold lysis buffer, complexes were solubilized in sample buffer, boiled, and resolved by SDS-PAGE. Proteins were transferred onto nitrocellulose membranes and immunoblotted using the SNAP-ID system (Millipore) following manufacturer's recommendations. Immunoblots were revealed using ECL (Millipore) and exposed to Fusion FX7 CCD camera (Vilber Lourmat). When required, densitometry of several experiments was done using ImageJ software and statistical analysis performed. For cross-linking experiments, Dimethyl pimelimidate (DMP, Thermo Fisher Scientific, Brebières, France) was added to the cleared cell lysates (1 h at room temperature) and the cross-linking reaction was stopped by addition of Tris (40 mM, pH 8) in the lysates before immunoprecipitation. For GST-binding assays, GST-fusion proteins adsorbed on glutathione-Sepharose beads [16] were incubated in cleared cell lysates for 2 hours at 4°C, washed in cold lysis buffer, and were processed for Western Blotting as described above. For Far Western Blotting, the prey proteins (Flag-ArgBP2 WT or P1) were precipitated from HEK293T cells, resolved by SDS-PAGE, and transferred to nitrocellulose membranes as in standard Western Blot. Then membranes were blocked in PBST (Phosphate-buffered saline, 5% milk, 0.05% Tween-20) for 1 hour and probed at 4°C o/n with 10 µg of purified bait (GST or GST-ArgBP2 fusion proteins) diluted in protein-binding buffer (100 mM NaCl, 20 mM Tris pH 7.6, 0.5 mM EDTA, 10% glycerol, 0.1% Tween-20, 2% milk, 1 mM DTT). The bait proteins were detected with HRP-conjugated anti-GST antibodies diluted at 1:500 in PBST.

### FPLC

Cell lysates containing 5 mg of protein were filtered through a 0.2 µm filter, and concentrated to a volume of 150 µl using Centricon Plus-20 (Millipore). The samples were applied to a Superdex 200 HR 10/30 column, which was equilibrated with Phosphate-buffered saline/0.1% Triton X-100, pH 7.5. Chromatography was run at 0.5 ml/min using a FPLC phamacia LKB and fractions of 0.5 mL were collected. Fractions were subjected to immunoprecipitation using anti-ArgBP2 (C1) antibodies followed by Western blot. The column was calibrated using the LMW gel filtration calibration kit (GE Healthcare).

### BRET (Bioluminescence Resonance Energy Transfer) assay

The pRluc expression vector was defined as BRET donor and pEYFP defined as BRET acceptor. We used a constant amount of BRET donor and increasing amounts of BRET acceptor. Empty pEYFP vector was used to equalize DNA amounts in each sample. Cells were transfected in 12-well culture plates with 1 µg of total plasmid DNA. 24 h later, cells were harvested and distributed in a white 96-well microplate (10^5^ cells/well). On the following day, cell media was replaced by Opti-MEM, and the cell-permeable Rluc substrate Coelentherazin-h (Promega) was added to a final concentration of 5 µM, 15 min before acquisition, using a TRISTAR, with signals detection set at 470–490 nm (donor) and 520–540 nm (acceptor) windows. To assess signal variation, BRET values were determined using the following equation, expressed in mBu (milli-BRET unit): (530 nm acceptor signal/480 nm donor signal – E_0_) ×1000, where E_0_ corresponds to the ratio 530 nm acceptor signal/480 nm donor signal obtained with the Rluc construct alone in the same experiment.

### Migration assay

The effect of ArgBP2 expression on MiaPaCa cell migration was determined using Multiwell Chemotaxis chambers (Neuro Probe, USA) with 8-µm pore polycarbonate filters (Nucleopore Track-Etch Membrane, GE Healthcare). The undersurface of filters was coated with 10 µg/mL of fibronectin. The lower tank was filled with DMEM containing 0.1% BSA and the membrane was placed on the chamber. Cells (50.10^3^) in DMEM containing 0.1% BSA were seeded on the top side of perforated filters. Following incubation (7 h), non migratory cells on the upper surface of the filter were wiped with a cotton swab. Cells that migrated to the lower surface of the filter were fixed and stained with Coomassie blue. Haptotaxis was determined by counting cells in 5 random microscopic fields (magnification, ×100) per well. Migration results were expressed as the average number of migrating cells per microscopic field. Assays were repeated more than three times and statistic was applied.

### Spreading assay

24-well plates were coated overnight at 4°C with 250 µL of fibronectin at 10 µg/ml. Coated wells were washed twice with PBS and were blocked with 0.5% BSA in PBS for 30 min. Single cell suspensions (50,000 cells in 0.5 ml) were seeded onto substratum-coated wells and allowed to adhere for 2 h at 37°C. Cell areas were measured microscopically using ImageJ software. Assays were repeated more than three times and statistic was applied.

### PLA (Proximity ligation assay) and immunofluorescence

PLA experiments were done in NIH3T3 and BxPC3 cells. 24 h after transfection (expression plasmids or siRNAs), cells were seeded on glass coverslips for an additional 24 h before performing PLA, and control immunofluorescence (Figure S1). Then cells were washed twice in PBS and incubated 10 minutes in PBS containing 4% paraformaldehyde. After being washed in PBS, cells were treated 20 minutes in PBS/50 mM NH4Cl, washed twice, permeabilized 3 min in PBS/0.2% Triton X-100 and washed twice. Protein-protein interactions were observed using the PLA technology (Duolink) according to manufacturer's recommendations (Olink). Expression of each transfected protein was verified by immunofluorescence as described previously [20]. Preparations were mounted using Prolong Gold antifade reagent (Invitrogen) and examined with Nikon microscope Eclipse 90I.

### Statistical Analysis

Microsoft Excel was used for statistical analysis. Values from independent experiments were compared using Student's t test and were expressed as mean ± standard deviation.

## Results

### ArgBP2 interacts with itself

**Figure 1:**
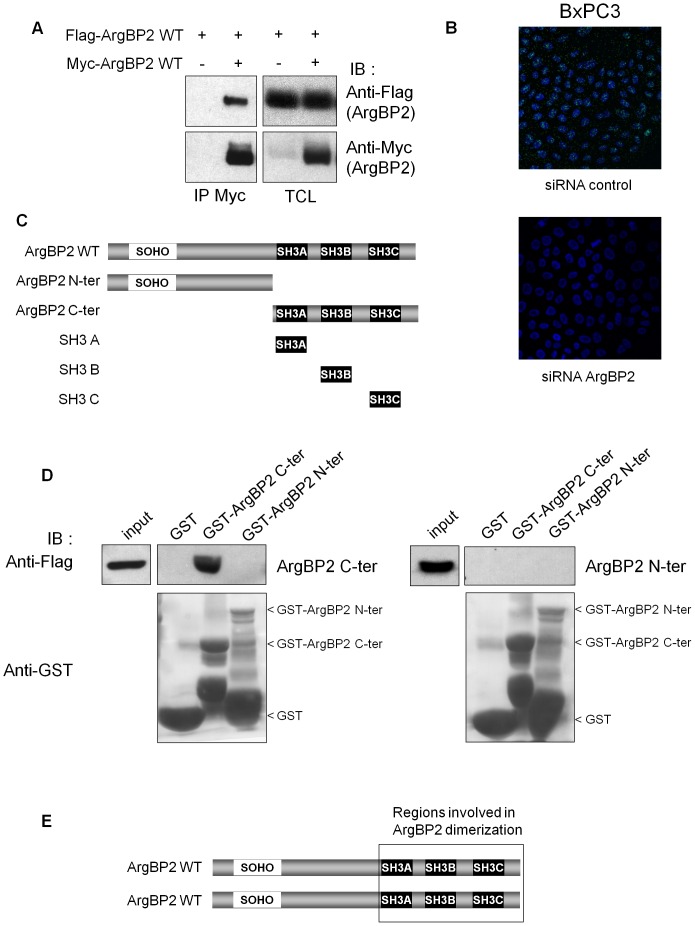
Oligomerization of ArgBP2. **A**) HEK293T cells were transfected with myc-ArgBP2 and flag-ArgBP2 expressing vectors as indicated. Lysates were subjected to immunoprecipitation (IP) with an anti-Myc antibody and membranes were first immunoblotted (IB) with an anti-Flag antibody. After stripping, membranes were blotted with an anti-Myc antibody to control the amount of precipitated material. The expression of the different constructs was controlled by blotting total cell lysates (TCL) with the corresponding antibodies. **B**) PLA study of endogenous ArgBP2 self-interaction in BxPC3 cells. Cells were transfected with ArgBP2 specific siRNA or control siRNA (48 h before) and anti-ArgBP2 monoclonal antibody (C1) conjugated to positive PLA probe was mixed with the same antibody conjugated to negative PLA probe to perform PLA experiments. **C**) Schematic representation of ArgBP2 indicating the different deletion mutants used. **D**) Lysates from HEK293T cells expressing Flag-tagged ArgBP2 N-terminal (ArgBP2 N-ter) or C-terminal (ArgBP2 C-ter) part (see Fig. B) were subjected to GST pull-down with N-terminal and C-terminal parts of ArgBP2 as indicated. The upper parts of membranes were immunoblotted with anti-Flag (ArgBP2 N-terminal or C-terminal), and the lower part with anti-GST antibody. **E**) Scheme of ArgBP2 parts involved in its interaction.

To explore the potential oligomerization of ArgBP2 proteins we have performed co-immunoprecipitation experiments using myc tagged ArgBP2 and Flag tagged ArgBP2 co-expressing cells. As shown in Figure 1A (and Figure S2), we could observe that ArgBP2 efficiently interacted with itself. Next, we confirmed this property of ArgBP2 with endogenous protein. To this end, we used BxPC3 cells which express endogenous ArgBP2 [21] and performed PLA experiment with only one anti-ArgBP2 monoclonal antibody thereby generating positive signal only if at least two ArgBP2 molecules interact. As expected, we observed a specific PLA signal in these cells that disappeared when ArgBP2 specific siRNA was transfected (Figure 1B). ArgBP2 contains three SH3 domains in its C-terminus and the SoHo domain in its N-terminal part (Figure 1C) which could all take part in ArgBP2 oligomerization. In order to discriminate which of these domains mediat ArgBP2 oligomerization, we have used GST protein fused to the N-terminus or the C-terminus of ArgBP2 to precipitate the Flag tagged N-terminus (Figure 1D, right panel) and C-terminus of ArgBP2 (Figure 1D, left panel). We observed that only the C-terminal part of ArgBP2 was able to interact with another C-terminal part. As the GST-ArgBP2 N-ter fusion did not bind to ArgBP2 N-ter or C-ter, we have controlled that it was functional by its ability to precipitate Flotillin, a known ArgBP2-Nter interacting protein (Figure S3). Hence, oligomerization of ArgBP2 involved one or several of its SH3 domains that bound to one or several proline motifs located in its C-terminus too (Figure 1E).

### Characterization of ArgBP2 oligomers

**Figure 2:**
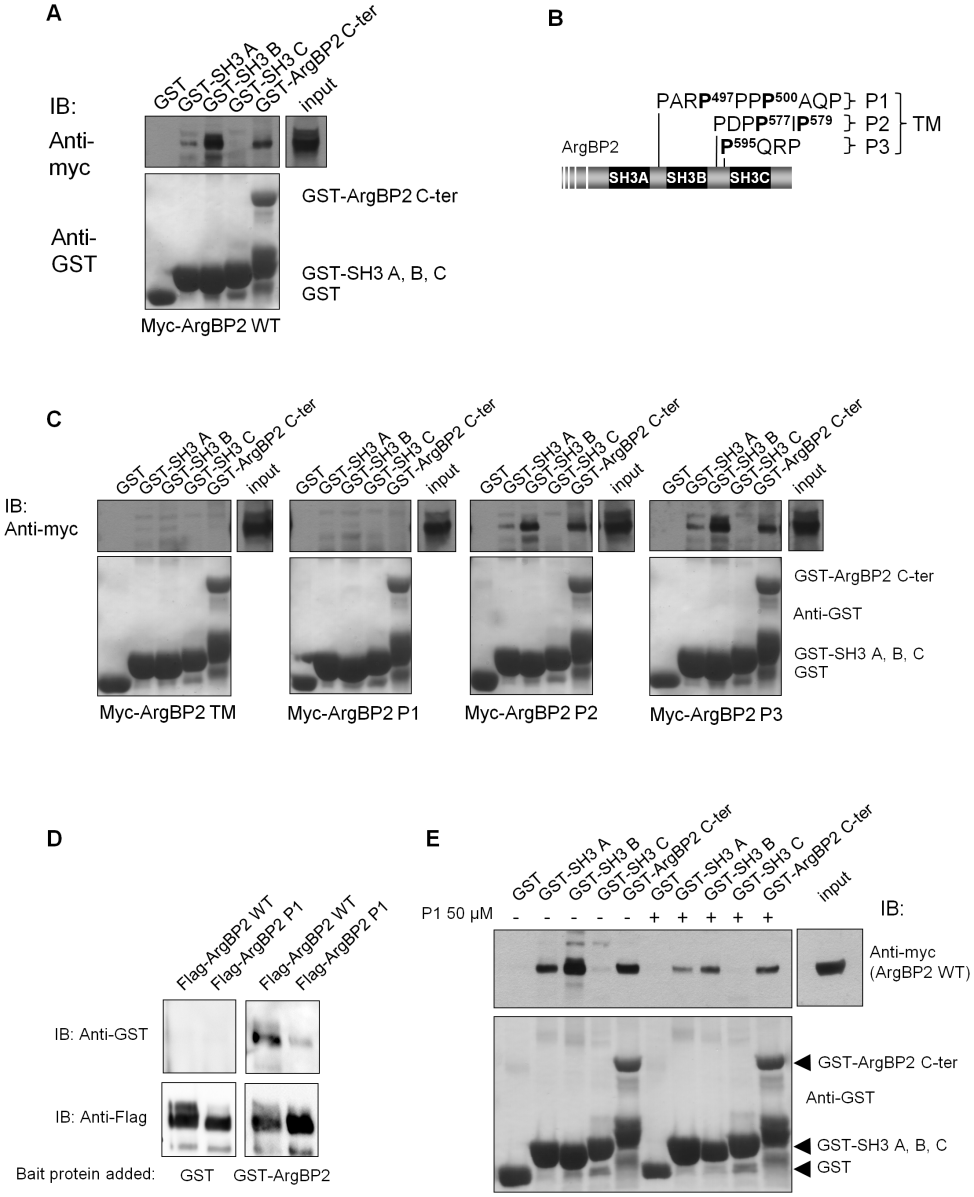
The interaction takes place between the SH3 B domain of one ArgBP2 protein and a proline motif of another one. **A**) Lysates from cells expressing myc-ArgBP2 were subjected to GST pull-down with different SH3 domains of ArgBP2. **B**) Schematic representation of ArgBP2 showing the three clusters of proline motifs contained in the C-terminal part of ArgBP2. Three mutants of ArgBP2 were generated in which the important prolines residues of each clusters were changed to alanines (ArgBP2 P1, ArgBP2 P2 and ArgBP2 P3). A mutant owning the triple mutation was generated (ArgBP2 TM). Mutated prolines are indicated in bold. **C**) Lysates from HEK293T cells transfected with proline clusters mutants of ArgBP2, were subjected to GST pull-down with different SH3 domains of ArgBP2 as indicated. The upper parts were immunoblotted (IB) with an anti-myc antibody (ArgBP2) and the lower parts with an anti-GST antibody. **D**) Far western blot of ArgBP2 WT and P1. Lysates from cells expressing Flag tagged ArgBP2 WT or P1 were probed with GST or GST-ArgBP2 fusion protein. Membranes were then revealed using anti-GST immunoblot. **E**) GST pull-down of myc-ArgBP2 WT using GST fused to SH3 domains or C-terminal part of ArgBP2 (as in A) in presence or not of 50 µM of P1 peptide.

To confirm the hypothesis that one or several SH3 domains mediate the oligomerization of ArgBP2, we have tested the ability of each single SH3 domains fused to GST (Figure 1B) to bind to full length ArgBP2 expressed in HEK-293T cells. We observed that, despite a weak interaction through the SH3 A domain, the SH3 B domain of ArgBP2 was the major binding module (Figure 2A). SH3 domains usually bind “PXXP” type proline motifs. The C-terminal part of ArgBP2 contains three proline rich clusters (P1, P2 and P3) containing one or several of this kind of proline motif (schematized in Figure 2B). To determine which one of these three proline clusters was involved in ArgBP2 self-interaction, we generated ArgBP2 mutant proteins in which the proline residues included in proline motifs of each cluster (P497 and P500 for ArgBP2 P1, P577 and P579 for ArgBP2 P2, and P595 for ArgBP2 P3) were individually (ArgBP2 P1, P2, and P3) or simultaneously (ArgBP2 triple mutant: TM) changed to alanine residues, thereby preventing their recognition by any SH3 domain. Subsequently, we have studied the ability of each GST-fused SH3 domains of ArgBP2 to bind to each proline mutants proteins. As expected, the simultaneous mutation of the three clusters of proline motifs (ArgBP2 TM) totally abolished ArgBP2 recognition by GST-ArgBP2 SH3 B and SH3 A, as well as by the C-terminal part containing all 3 SH3s together (Figure 2C). Importantly, whereas P2 and P3 mutants were still able to bind efficiently to SH3 A and B, the P1 mutant was completely unable to bind to both SH3 domains. Thus, we could establish that the ArgBP2/ArgBP2 interaction took place mainly between the SH3 B domain of one molecule and the proline cluster PARP^497^PPP^500^ (P1) contained in another one. To validate the direct and physical interaction between two ArgBP2 proteins, and to avoid any contribution of other ArgBP2 associated proteins, we performed Far-Western blots experiments using GST-fused to wild-type ArgBP2 as a first probe. Wild-type ArgBP2 precipitated from HEK293T cells, but not ArgBP2 P1 mutant, was recognized by GST-ArgBP2 (Figure 2D). This data confirmed the direct physical association of ArgBP2 with itself and demonstrated the essential role played by the P1 cluster. To further validate the molecular mechanism of ArgBP2 oligomerization, we used a peptide whose sequence corresponds to the P1 proline stretch as a binding competitor. As expected, the use of this peptide, during GST pull-down, strongly impaired the interaction between SH3 B/A and ArgBP2 WT protein (Figure 2E). Curiously, the effect of the P1 peptide was not complete and residual interactions remained. This observation suggests that the P1 stretch, in the context of full ArgBP2 protein, benefited from an imposed structural conformation that the peptide alone could not spontaneously form.

### ArgBP2 oligomerization in a cellular context

**Figure 3:**
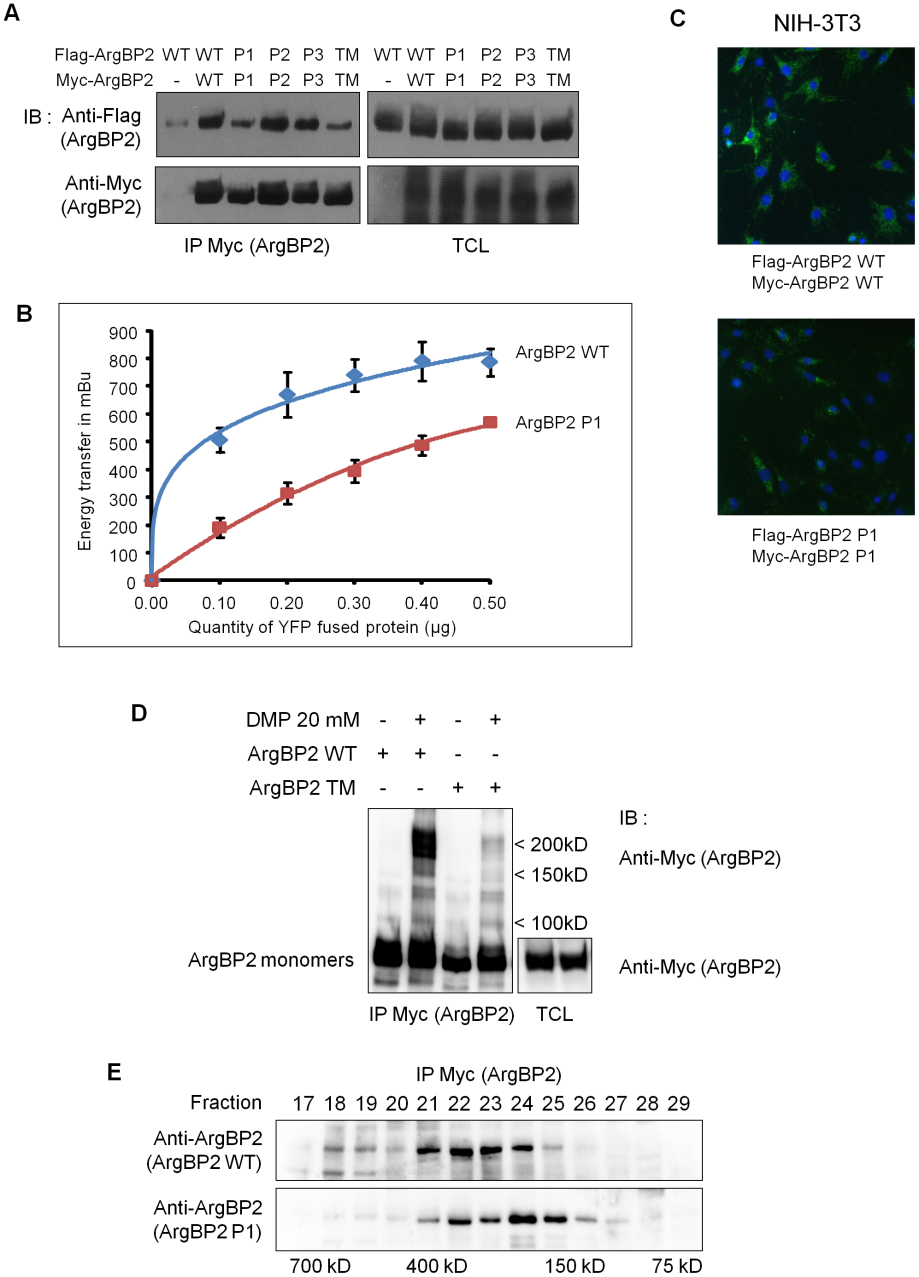
Mutation of P1 cluster of proline motifs affects ArgBP2's oligomerization. **A**) Lysates from HEK293T cells, expressing myc and Flag tagged version of WT or mutants ArgBP2, were subjected to immunoprecipitation (IP) with an anti-Myc antibody and membranes were immunoblotted (IB) with an anti-Flag antibody. After stripping, membranes were blotted with an anti-Myc antibody to control the amount of precipitated material. The expression of the different constructs was controlled by blotting total cell lysates (TCL) with the corresponding antibodies. **B**) BRET assay using ArgBP2 WT or P1 mutant. A constant amount of Luciferase fused to ArgBP2 WT or P1 was co-transfected in HEK293T cells with increasing amount of EYFP fused ArgBP2 WT or P1 respectively. BRET signal has been calculated as described in experimental procedures and one representative experiment is shown. **C**) PLA assay for ArgBP2 oligomerization using NIH-3T3 cells transfected with Myc and Flag tagged ArgBP2 WT, or P1 mutant. **D**) Lysates from HEK293T cells transfected with Myc tagged ArgBP2 WT or ArgBP2 TM were treated (+ DMP) or not (- DMP) with a cross-linking reagent (Dimethyl pimelimidate, DMP). Then, lysates were subjected to immunoprecipitation with an anti-Myc antibody and membranes were immunoblotted with an anti-Myc antibody. Expression of both constructs was controlled by blotting total cell lysates (TCL) with an anti-Myc antibody. **E**) Gel filtration assay. Macromolecular complexes from lysates of HEK293T cells expressing either ArgBP2 WT or ArgBP2 P1 mutant, were separated by FPLC as described in experimental procedures. Fractions were subjected to immunoprecipitation using an anti-Myc antibody and membranes were immunoblotted using anti-ArgBP2 antibody.

We next aimed to confirm the ArgBP2 oligomerization and the role of the P1 proline cluster at the cellular level. Therefore, we performed co-immunoprecipitation experiments with wild-type and mutants ArgBP2 proteins. Consistent with our *in vitro* results, inactivation of P1 cluster strongly reduced the ArgBP2/ArgBP2 interaction, whereas alteration of P2 or P3 clusters had no impact (Figure 3A). Moreover, the simultaneous mutation of P1, P2 and P3 clusters had the same effect than P1 mutation alone, indicating that only P1 proline cluster was essential for ArgBP2 oligomerization *in cellulo*. This observation was confirmed in living cells using BRET (Figure 3B) and PLA (Figure 3C) techniques. Intriguingly, in all these experiments, residual self-interaction between P1 or TM mutant ArgBP2 proteins subsisted. This observation suggests that despite the major role played by the P1 cluster in the direct oligomerization of ArgBP2, in cellular context, some secondary and indirect interactions (probably some of the ArgBP2 binding partners) could enable residual ArgBP2/ArgBP2 association.

As a scaffold protein, ArgBP2 primarily interacts with other proteins and forms multi-molecular complexes. We have performed cross-linking experiments (Figure 3D) and gel filtration assays (Figure 3E) to visualize the ArgBP2 containing complexes. We could detect ArgBP2 WT in a complex of 150 KDa which could correspond to ArgBP2 dimers (Figure 3D). This complex almost disappeared with the P1 mutant. Other major ArgBP2 containing complexes, in the range of 200 kDa and which could correspond to ArgBP2 homotrimers and to heteromers of ArgBP2 with some of its associated proteins, was also observed and was strongly reduced with the P1 mutation (Figure 3D). These results were confirmed by gel-filtration experiments where mutating the P1 cluster impaired, partially, the ability of ArgBP2 to form high molecular weight complexes (Figure 3E). Hence, oligomerization of ArgBP2 favored the formation of bigger molecular complexes.

### ArgBP2 oligomerization regulates its interaction with its partners

**Figure 4:**
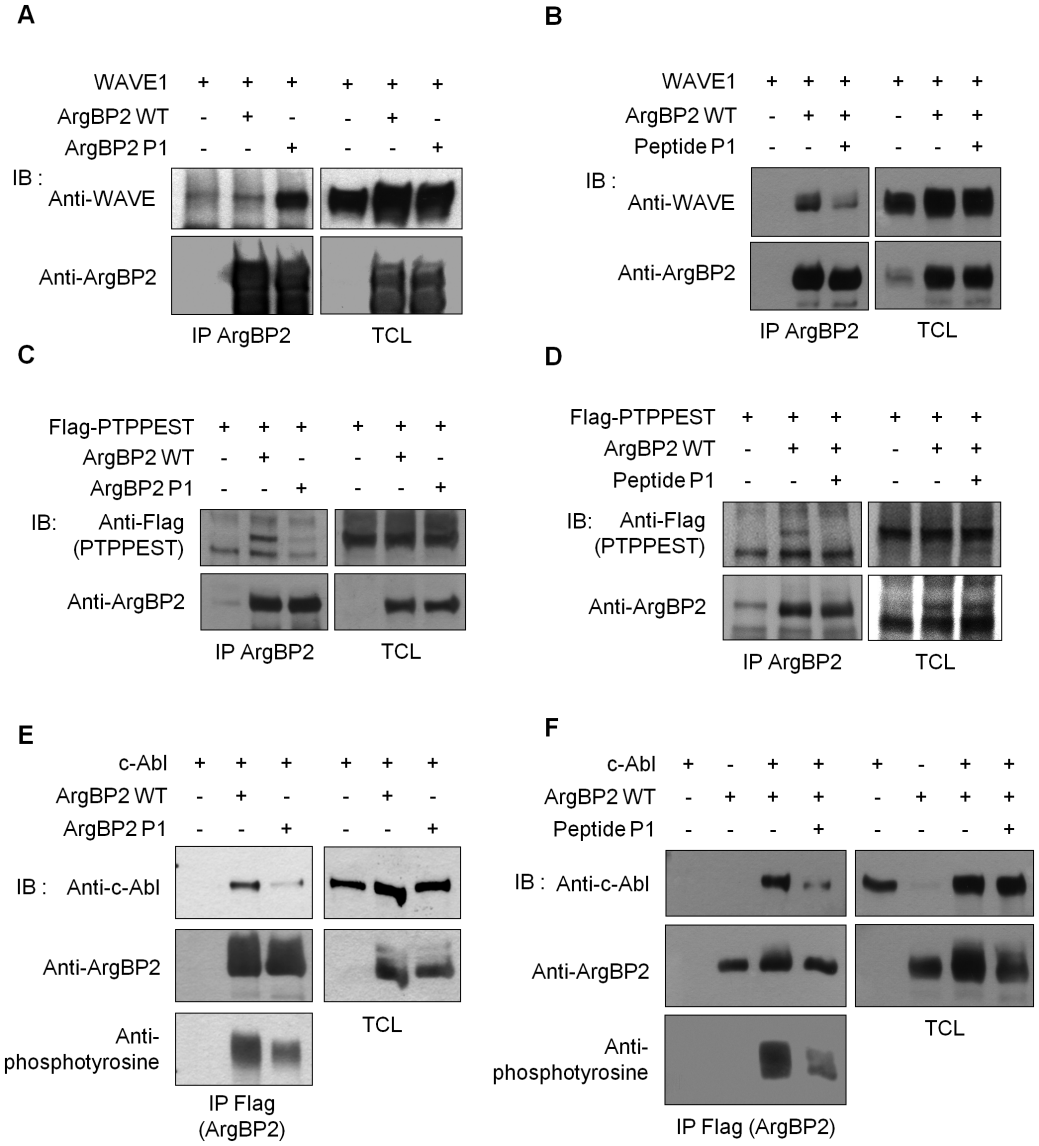
ArgBP2 oligomerization can modulate its binding to some of its partners. **A**) Co-immunoprecipitation of ArgBP2 WT or ArgBP2 P1 with WAVE1. Lysates from HEK293T cells, transfected as indicated, were subjected to immunoprecipitation (IP) using an anti-ArgBP2 antibody. Membranes were first immunoblotted (IB) with an anti-WAVE antibody then, after stripping, membranes were blotted with an anti-ArgBP2 antibody to control the amount of precipitated material. Expression of all proteins was controlled by blotting total cell lysates (TCL) with the corresponding antibodies. **B**) Effect of the P1 peptide upon the ArgBP2/WAVE1 co-immunoprecipitation. Lysates from cells expressing WAVE1 and ArgBP2 WT were treated or not with the P1 peptide prior to IP using an anti-ArgBP2 antibody. Membranes were first immunoblotted with an anti-WAVE antibody then, after stripping, with an anti-ArgBP2 antibody to control the amount of precipitated material. Proteins expressions were controlled by blotting TCL with the corresponding antibodies. **C**) As in **A**), co-immunoprecipitation of ArgBP2 WT or P1 mutant with PTP-PEST. **D**) As in **B**), effect of P1 peptide upon the ArgBP2/PTP-PEST co-immunoprecipitation. **E**) As in **A**), co-immunoprecipitation of ArgBP2 WT or P1 mutant with c-Abl, and resulting phosphorylation of ArgBP2. **F**) As in **B**), effect of P1 peptide upon the ArgBP2/c-Abl co-immunoprecipitation and ArgBP2 phosphorylation.

ArgBP2 is an adaptor protein and its functions are highly dependent on its binding to its associated proteins. Therefore we have studied how oligomerization of ArgBP2 may modulate its interactions with some of its known interacting partners. We compared the ability of ArgBP2 WT and of ArgBP2 P1 mutant to bind to some of them and we have explored the impact of P1 peptide on these associations. Inhibition of ArgBP2 self-association greatly enhanced the binding to WAVE1 (Figure 4A), indicating that WAVE1 preferentially associated with monomers of ArgBP2. The presence of P1 peptide greatly reduced the WAVE1/ArgBP2 association (Figure 4B). On the contrary, inhibition of ArgBP2 oligomerization greatly reduced its binding to PTP-PEST (Figure 4C) and c-Abl (Figure 4E), meaning that both PTP-PEST and c-Abl preferentially associated with oligomers of ArgBP2. Using P1 peptide efficiently inhibited the association of ArgBP2 with both PTP-PEST (Figure 4D) and c-Abl (Figure 4F). Logically, the decreased association of ArgBP2 P1 with c-Abl also resulted in a reduced phosphorylation of ArgBP2 P1 (Figures 4E and 4F).

### ArgBP2 oligomerization is regulated by c-Abl mediated phosphorylation

**Figure 5:**
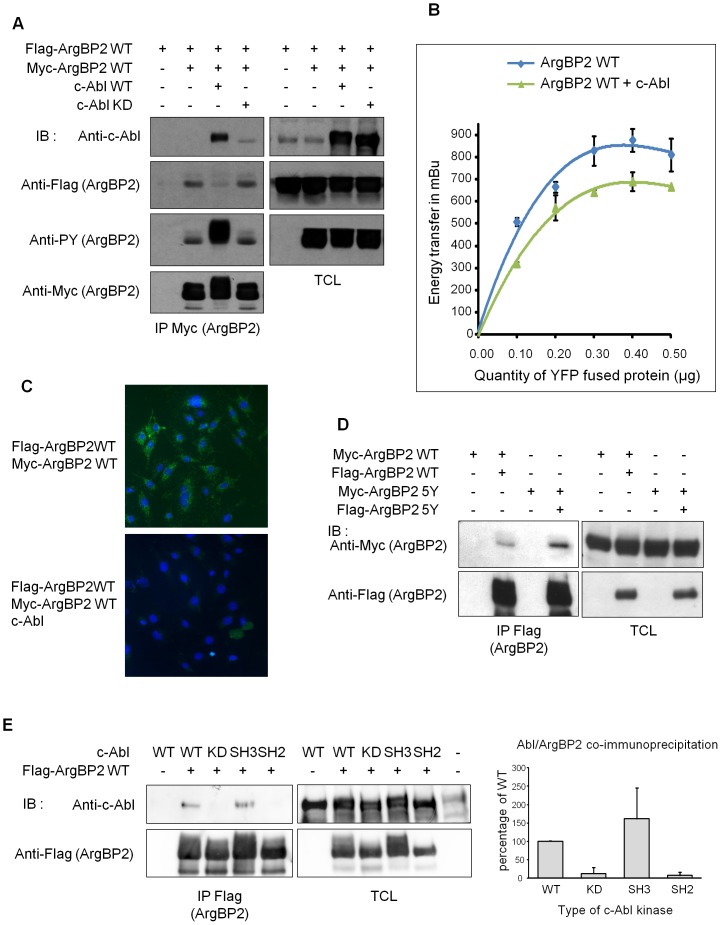
Phosphorylation of ArgBP2 by c-Abl regulates its oligomerization. **A**) HEK293T cells were transfected with Myc and Flag tagged ArgBP2, wild type c-Abl (WT) and kinase-inactive c-Abl (KD) expressing vectors, as indicated. Lysates were subjected to immunoprecipitation (IP) using an anti-Myc antibody and membranes were immunoblotted (IB) using an anti-Flag (ArgBP2) antibody at ArgBP2 level, and with an anti-c-Abl antibody at c-Abl level. After stripping, membranes were blotted with an anti-phosphotyrosine (PY) antibody to estimate the phosphorylation of ArgBP2 and, in a third time, with an anti-Myc antibody (to control the amount of precipitated material). Expression of all proteins was controlled by blotting total cell lysates (TCL) with the corresponding antibodies. **B**) BRET analysis of the effect of c-Abl upon ArgBP2 self-association. HEK293T cells were transfected with a constant amount of Luciferase fused to ArgBP2 WT and an increasing amount of EYFP fused ArgBP2 WT. BRET signal has been calculated as described in experimental procedures. **C**) PLA study of ArgBP2 WT oligomerization in presence or not of c-Abl, using NIH-3T3 cells transfected with Myc and Flag tagged ArgBP2 WT with or without c-Abl expressing vectors. **D**) HEK293T cells were transfected with myc and flag tagged ArgBP2, wild type (WT) or five tyrosines mutant (5Y). Lysates were subjected to IP with an anti-Flag antibody and membranes were blotted using anti-Myc antibody. After stripping, membranes were blotted using anti-Flag antibody to control the amount of precipitated material. The expression of the different constructs was controlled by blotting TCL with the corresponding antibodies. **E**) Lysates from 293T cells expressing Flag tagged ArgBP2 WT together with c-Abl WT, the kinase deficient (KD), the SH2 domain mutant (SH2), or the SH3 domain mutant (SH3), were subjected to IP using an anti-Flag antibody to precipitate ArgBP2. The amount of co-precipitated c-Abl was revealed by blotting the upper part of the membrane with an anti-c-Abl antibody, and the amount of precipitated ArgBP2 was controlled by blotting the lower part with an anti-Flag antibody. The expression of all constructs was verified in TCLs. **E**) (right) Graphical representation of pooled data from three independent experiments.

Because ArgBP2 association with both the kinase c-Abl and the phosphatase PTP-PEST was regulated by its oligomerization we wondered if, inversely, phosphorylation state of ArgBP2 could modulate its oligomerization. Using c-Abl WT and KD (kinase deficient), we observed that tyrosine phosphorylation of ArgBP2 strongly impaired the co-immunoprecipitation of ArgBP2 with itself (Figure 5A and Figure S2). Moreover, c-Abl bound more avidly to phosphorylated ArgBP2 than to unphosphorylated ArgBP2 (Figure 5A). Importantly, this effect of c-Abl mediated phosphorylation upon ArgBP2 self-association was confirmed in living cells both by BRET (Figure 5B) and by PLA (Figure 5C).

In addition to the sole effect of tyrosine phosphorylation of ArgBP2, the increased ArgBP2/c-Abl interaction could also contribute to the dissociation of ArgBP2 oligomers. To discriminate between the role of tyrosine phosphorylation of ArgBP2 and of c-Abl binding, we performed complementary experiments. Among the 30 tyrosine residues of ArgBP2, five have high probability of being targeted by c-Abl mediated phosphorylation (Figure S4). Therefore, we have generated a mutant ArgBP2 protein in which all these five tyrosine residues are changed to non-phosphorylable phenylalanine residues (ArgBP2 5Y). As expected, co-immunoprecipitation of ArgBP2 5Y was higher than co-immunoprecipitation of ArgBP2 WT (Figure 5D), confirming that tyrosine phosphorylation of ArgBP2 reduced its self-association. Finally, we have used several c-Abl mutants (KD, SH2, and SH3 mutants) in order to clarify the c-Abl/ArgBP2 mode of interaction (Figure 5E). As previously shown, c-Abl KD bound less to ArgBP2 than c-Abl WT. Interestingly, like c-Abl KD, c-Abl SH2 mutant also bound poorly to ArgBP2 (Figure 5E). On the contrary, SH3 mutant of c-Abl bound to ArgBP2 as efficiently as WT c-Abl (Figure 5E). Hence, the increased c-Abl/ArgBP2 interaction was mainly dependent on the binding of the SH2 domain of c-Abl to phosphorylated tyrosines of ArgBP2.

### Oligomerization dependent cellular functions of ArgBP2

**Figure 6:**
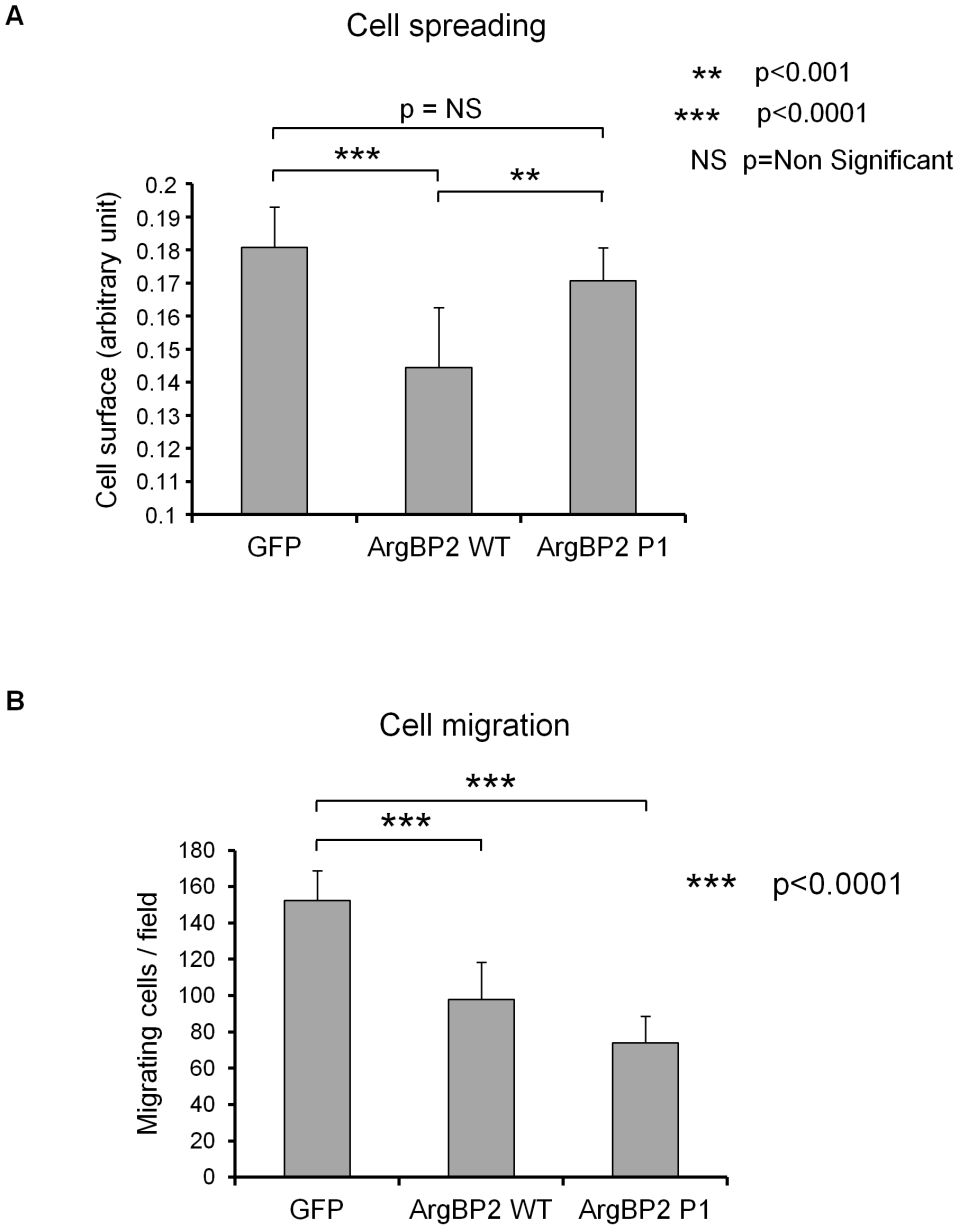
Role of ArgBP2 oligomerization regarding cell spreading and migration. **A**) Miapaca-2 cells stably expressing ArgBP2 WT, ArgBP2 P1 mutant, or GFP as control, were seeded onto fibronectin coated culture plates. After two hours, cells were fixed and stained using Coomassie blue. Ten pictures of each were taken and the size of each cell has been evaluated using ImageJ software (NIH). Three independent experiments were used to generate the data which are represented as mean +/- SD and Student T test has been performed to compare GFP with ArgBP2 WT and ArgBP2 WT with ArgBP2 P1 expressing cells. **B**) Miapaca-2 cells stably expressing ArgBP2 WT, ArgBP2 P1 mutant, or GFP as control, were subjected to cell migration assays in Boyden chambers during 7 hours. Migrating cells were then fixed and stained using Coomassie blue. Each experiment was done in triplicate and five random pictures were taken from each well. Three independent experiments were pulled together and cell migration is expressed as a percentage of control cells (GFP) migration +/- SD and student T test has been done to compare GFP with ArgBP2 WT and GFP with ArgBP2 P1 expressing cells.

Considering that oligomerization of ArgBP2 can regulate some of its interactions, we assumed that this property of ArgBP2 could regulate some of its cellular functions. Therefore, we compared ArgBP2 WT and ArgBP2 P1 expressing MiaPaCa2 pancreatic cancer cells in order to study what is the biological relevance of ArgBP2 oligomerization. Expression of ArgBP2 WT in these cells is known to inhibit cell adhesion, cell spreading and cell migration [21]. We could observe that, contrary to ArgBP2 WT, ArgBP2 P1 mutant was unable to decrease MiaPaCa2 cells spreading (Figure 6A). Therefore, self-interaction of ArgBP2 was required for the ArgBP2 mediated inhibition of pancreatic cancer cells spreading. Surprisingly, ArgBP2 P1 mutant was as efficient as ArgBP2 WT in inhibiting MiaPaCa2 cells' migration (Figure 6B). Hence, the control of pancreatic cancer cells migration by ArgBP2 was not dependent on its oligomerization capability.

## Discussion

ArgBP2 and other members of the SoHo family are adaptor proteins involved in the regulation of signalling events and actin cytoskeleton functions. They have been involved in RTKs signalling [3], [29], in a variety of intracellular signals [10], [17], [19], [30], [31], as well as in the modulation of cell adhesion, spreading and migration [7], [11], [18], [19], [20], [21], [30], [32]. Their important roles as modulators, and sometime actors, of these central biological processes have been revealed by their deficiencies in diverse human pathologies including cancers [2]. Therefore, to understand how these proteins function and how they are regulated turns out to be of a primary interest. Part of their mechanisms of action has been revealed by the identification of a still growing number of molecular partners, many of them being shared between ArgBP2 CAP and vinexin, such as vinculin, flotillin, c-Cbl and c-Abl [2]. However, the mechanisms controlling the way these proteins preferentially interact with a set of their molecular partners, at a given time and space in cell, remain largely unclear.

One of these regulatory mechanisms involves alternative splicing. Indeed, different isoforms of ArgBP2 (nArgBP2, ArgBP2 alpha, beta, gamma) are expressed depending on the cell/tissue compartment, conferring new topology specific functions to the protein [13], [14], [17], [18]. This regulatory mechanism is shared by other members of this protein family [7], [33]. Another major regulatory mechanism of SoHo proteins functions involves their tyrosine and serine/threonine phosphorylation [13], [17], [21], [34], [35]. We assume that phosphorylation of ArgBP2 facilitates the recruitment of SH2 or PTB domain containing protein, such as c-Abl and c-Cbl respectively ([16] and Figure 5E). But, phosphorylation of ArgBP2 also regulates its interaction with partners devoid of any phospho-residue recognition domain. Hence, it is mostly probable that ArgBP2 phosphorylation induces conformational changes resulting in the exposure and hiding of distinct interaction modules. By showing that phosphorylation of ArgBP2 regulates its oligomerization, our results explain part of this mechanism.

One remaining critical point regarding the adaptive function of ArgBP2 is that different ArgBP2 interacting proteins share the same SH3(s) domain(s) of ArgBP2. Consequently, multiple partners may compete with each other for binding to ArgBP2. For example, as c-Abl binds to SH3 A and C of ArgBP2 [13] while WAVE1 binds to SH3 A and B [21], c-Abl and WAVE1 compete for binding to SH3 B domain. Hence, one possibility to overcome this problem is that ArgBP2 can form oligomers. Using a panel of *in vitro* and *in cellulo* techniques, we were able to confirm that ArgBP2 interacts with itself to form oligomers, thereby multiplying the number and variety of ArgBP2 partners contained in one complex.

We next aimed to determine how this oligomerization takes place. Because there is no known oligomerization domain in ArgBP2, we firstly hypothesized that one or several SH3 domains of ArgBP2 could bind to one or several of its proline motifs. We could show that this is indeed the case but, surprisingly, the involved proline motif was not located within the major proline riche region of ArgBP2 (between amino-acids 213 and 228) but within a smaller proline riche region located between the first and second SH3 domains. Moreover, the fact that oligomerization of ArgBP2 occurred mainly via its SH3 B domain implies that self-interaction of ArgBP2 would lead to a molecular complex where most of the N-terminal part and the SH3 A and C of ArgBP2 are fully accessible, whereas the SH3 B is already engaged. Hence, oligomerization of ArgBP2 would generate a molecular platform with an increased number of available SH3 A and SH3 C domains and a decreased number of available SH3 B domains. This hypothesis is supported by the fact that WAVE1, which interacts with ArgBP2 via the SH3 A and B [21], bound preferentially monomers of ArgBP2 (Figure 4A), whereas c-Abl and PTP-PEST, which can also bind to other SH3s, had a better affinity for oligomers of ArgBP2 (Figures 4C and 4E). Intriguingly, whereas WAVE1 had a better affinity for ArgBP2 P1 mutant (Figure 4A) the use of the P1 peptide reduced this WAVE1/ArgBP2 interaction. In fact, considering that WAVE1 binds principally to SH3 B of ArgBP2 [21] the use of this peptide must saturate the SH3 B thereby impeding the binding of WAVE1 or any other SH3B interacting protein. Finally, it is important to notice that interaction of ArgBP2 with some of its associated proteins, like CIP4, was not influenced by its oligomerization (Figure S5).

As phosphorylation of ArgBP2 is well known to regulate some of its functions [11], [13], [16], [20], [21], we assumed that this post-translational modification of ArgBP2 could also alter its oligomerization. Indeed, we could observe that ArgBP2 tyrosine phosphorylation by c-Abl strongly inhibited ArgBP2 self-association (Figure 5A). Accordingly, oligomerization of phospho-deficient ArgBP2 5Y mutant was stronger than oligomerization of ArgBP2 WT (Figure 5D). Importantly, we could validate that phosphorylation induced destabilization of ArgBP2 oligomers is one regulatory mechanism of ArgBP2 interactions. Indeed, lack of binding of PTP-PEST to the oligomerization deficient ArgBP2 P1 mutant mimicked the effect of ArgBP2 phosphorylation regarding this interaction [21]. This result tends to prove that despite its role in recruiting phosphotyrosine recognition domain containing proteins, phosphorylation of ArgBP2 also regulates its binding to its associated partners by destabilizing ArgBP2 oligomers.

Unlike ArgBP2 WT, ArgBP2 P1 mutant was unable to inhibit pancreatic cancer cells spreading whereas it was still able to inhibit their migration (Figure 6). Importantly, whereas cell spreading mainly depends on membrane protrusion supported by actin filaments polymerization and establishment of new focal adhesions, cell migration further requires retraction at the cell's rear, contraction of the cell body, actin filaments depolymerization and cell detachment [36]. Hence, it seems that ArgBP2 must oligomerize to inhibit processes that are dependent on actin polymerization and adhesion formation (construction processes) whereas it obviously has the ability to inhibit actin depolymerization and/or de-adhesion dependent mechanisms (demolition processes) both as monomers and oligomers.

Functions endowed by other members of the SoHo family (CAP and vinexin) may be dependent on the same regulatory mechanism of self-association. Future investigations will shed light on this point. Undoubtedly, members from this family of proteins may also form hetero-oligomers. Indeed, we have used mass spectrometry in order to identify proteins contained in the cross-linked immunoprecipitated ArgBP2 complexes (data not shown) and found that CAP/Ponsin was one of the constituents. We could confirm this ArgBP2/CAP association by co-immunoprecipitation of the two proteins (Figure S6). Therefore, depending on their tissue expression profile, different members of the SoHo family expressed in a cell may collaborate by hetero-oligomerization to support a broader range of possible regulatory processes.

## Supporting Information

Figure S1 **Control immunofluorescence of NIH3T3 cells used for PLA assays.** (A) Control immunofluorescence corresponding to Figure 3C. Cells were stained with rabbit anti-Flag and mouse anti-Myc antibodies, followed by staining with the corresponding fluorescent secondary antibodies (Alexa-546 and 488). (B) Control immunofluorescence corresponding to Figure 5C. Cells were stained with rabbit anti-Flag and mouse anti-Myc antibodies, or with anti-Abl antibodies, followed by staining with the corresponding fluorescent secondary antibodies (Alexa-546 and 488).
(TIF)

Figure S2 **Co-immunoprecipitation of ArgBP2 with itself.** Lysates from 293T cells transfected as indicated were subjected to immunoprecipitation using an anti-Flag antiboby. Immunoprecipitated proteins (IP Flag), as well as total cell lysates (TCL), were analyzed by western blotting using the indicated antibodies.
(TIF)

Figure S3 **Control of the GST-ArgBP2 N-ter construct.** Flotillin is known to interact with ArgBP2 N-terminal part. As a positive control of the efficacy of the GST-ArgBP2 N-ter construct, the lysates from HEK293T cells expressing Flotillin were subjected to GST pull-down with N-terminal part of ArgBP2. The upper parts of filters were immunoblotted using anti-Flotillin antibody, and the lower part with anti-GST antibody.
(TIF)

Figure S4 Schematic representation of ArgBP2 indicating the position of the 5 tyrosine residues (Y50, Y59, Y164, Y175 and Y573, indicated by asterisks) contained in consensus sequences for c-Abl mediated phosphorylation.
(TIF)

Figure S5 **Co-immunoprecipitation of ArgBP2 WT and P1 with CIP4 protein.** 293T cells were transfected as indicated and lysates were subjected to immunoprecipitation (IP) using an anti-ArgBP2 antibody. Immunoprecipitated material and total cell lysates were immunoblotted (IB) using an anti-Flag antibody (CIP4) then, after stripping, with an anti-ArgBP2 antibody to control the amount of precipitated material.
(TIF)

Figure S6 **Co-immunoprecipitation of ArgBP2 with CAP/Ponsin.** 293T cells were transfected as indicated and lysates were subjected to immunoprecipitation (IP) using an anti-Myc (ArgBP2) antibody. Immunoprecipitated proteins, as well as cell lysates (TCL), were analyzed by western blot using an anti-Flag (CAP) antibody and, after stripping, with an anti-Myc (ArgBP2) antibody.
(TIF)

## References

[1] Kioka N, Ueda K, Amachi T (2002) Vinexin, CAP/ponsin, ArgBP2: a novel adaptor protein family regulating cytoskeletal organization and signal transduction. Cell Struct Funct 27: 1–7. 11937713 10.1247/csf.27.1

[2] Roignot J, Soubeyran P (2009) ArgBP2 and the SoHo family of adapter proteins in oncogenic diseases. Cell Adh Migr 3: 167–170. 19262174 10.4161/cam.3.2.7576 PMC2679877

[3] Baumann CA, Ribon V, Kanzaki M, Thurmond DC, Mora S, et al (2000) CAP defines a second signalling pathway required for insulin-stimulated glucose transport. Nature 407: 202–207. 11001060 10.1038/35025089

[4] Chang L, Adams RD, Saltiel AR (2002) The TC10-interacting protein CIP4/2 is required for insulin-stimulated Glut4 translocation in 3T3L1 adipocytes. Proc Natl Acad Sci U S A 99: 12835–12840. 12242347 10.1073/pnas.202495599 PMC130546

[5] Chiang SH, Baumann CA, Kanzaki M, Thurmond DC, Watson RT, et al (2001) Insulin-stimulated GLUT4 translocation requires the CAP-dependent activation of TC10. Nature 410: 944–948. 11309621 10.1038/35073608

[6] Breitsprecher D, Kiesewetter AK, Linkner J, Urbanke C, Resch GP, et al (2008) Clustering of VASP actively drives processive, WH2 domain-mediated actin filament elongation. EMBO J 27: 2943–2954. 18923426 10.1038/emboj.2008.211 PMC2585163

[7] Kioka N, Sakata S, Kawauchi T, Amachi T, Akiyama SK, et al (1999) Vinexin: a novel vinculin-binding protein with multiple SH3 domains enhances actin cytoskeletal organization. J Cell Biol 144: 59–69. 9885244 10.1083/jcb.144.1.59 PMC2148117

[8] Mitsushima M, Suwa A, Amachi T, Ueda K, Kioka N (2004) Extracellular signal-regulated kinase activated by epidermal growth factor and cell adhesion interacts with and phosphorylates vinexin. J Biol Chem 279: 34570–34577. 15184391 10.1074/jbc.M402304200

[9] Mitsushima M, Ueda K, Kioka N (2006) Vinexin beta regulates the phosphorylation of epidermal growth factor receptor on the cell surface. Genes Cells 11: 971–982. 16923119 10.1111/j.1365-2443.2006.00995.x

[10] Mizutani K, Ito H, Iwamoto I, Morishita R, Deguchi T, et al (2007) Essential roles of ERK-mediated phosphorylation of vinexin in cell spreading, migration and anchorage-independent growth. Oncogene 26: 7122–7131. 17486060 10.1038/sj.onc.1210512

[11] Cestra G, Toomre D, Chang S, De Camilli P (2005) The Abl/Arg substrate ArgBP2/nArgBP2 coordinates the function of multiple regulatory mechanisms converging on the actin cytoskeleton. Proc Natl Acad Sci U S A 102: 1731–1736. 15659545 10.1073/pnas.0409376102 PMC547834

[12] Ronty M, Taivainen A, Moza M, Kruh GD, Ehler E, et al (2005) Involvement of palladin and alpha-actinin in targeting of the Abl/Arg kinase adaptor ArgBP2 to the actin cytoskeleton. Exp Cell Res 310: 88–98. 16125169 10.1016/j.yexcr.2005.06.026

[13] Wang B, Golemis EA, Kruh GD (1997) ArgBP2, a multiple Src homology 3 domain-containing, Arg/Abl-interacting protein, is phosphorylated in v-Abl-transformed cells and localized in stress fibers and cardiocyte Z-disks. J Biol Chem 272: 17542–17550. 9211900 10.1074/jbc.272.28.17542

[14] Kawabe H, Hata Y, Takeuchi M, Ide N, Mizoguchi A, et al (1999) nArgBP2, a novel neural member of ponsin/ArgBP2/vinexin family that interacts with synapse-associated protein 90/postsynaptic density-95-associated protein (SAPAP). J Biol Chem 274: 30914–30918. 10521485 10.1074/jbc.274.43.30914

[15] Haglund K, Ivankovic-Dikic I, Shimokawa N, Kruh GD, Dikic I (2004) Recruitment of Pyk2 and Cbl to lipid rafts mediates signals important for actin reorganization in growing neurites. J Cell Sci 117: 2557–2568. 15128873 10.1242/jcs.01148

[16] Soubeyran P, Barac A, Szymkiewicz I, Dikic I (2003) Cbl-ArgBP2 complex mediates ubiquitination and degradation of c-Abl. Biochem J 370: 29–34. 12475393 10.1042/BJ20021539 PMC1223168

[17] Yuan ZQ, Kim D, Kaneko S, Sussman M, Bokoch GM, et al (2005) ArgBP2gamma interacts with Akt and p21-activated kinase-1 and promotes cell survival. J Biol Chem 280: 21483–21490. 15784622 10.1074/jbc.M500097200

[18] Murase K, Ito H, Kanoh H, Sudo K, Iwamoto I, et al (2012) Cell biological characterization of a multidomain adaptor protein, ArgBP2, in epithelial NMuMG cells, and identification of a novel short isoform. Med Mol Morphol 45: 22–28. 22431180 10.1007/s00795-010-0537-9

[19] Martin M, Geudens I, Bruyr J, Potente M, Bleuart A, et al (2013) PP2A regulatory subunit Balpha controls endothelial contractility and vessel lumen integrity via regulation of HDAC7. EMBO J 32: 2491–2503. 23955003 10.1038/emboj.2013.187 PMC3770954

[20] Roignot J, Taieb D, Suliman M, Dusetti NJ, Iovanna JL, et al (2009) CIP4 is a new ArgBP2 interacting protein that modulates the ArgBP2 mediated control of WAVE1 phosphorylation and cancer cell migration. Cancer Lett 288: 116–123. 19631450 10.1016/j.canlet.2009.06.030

[21] Taieb D, Roignot J, Andre F, Garcia S, Masson B, et al (2008) ArgBP2-dependent signaling regulates pancreatic cell migration, adhesion, and tumorigenicity. Cancer Res 68: 4588–4596. 18559503 10.1158/0008-5472.CAN-08-0958

[22] Suetsugu S, Yamazaki D, Kurisu S, Takenawa T (2003) Differential roles of WAVE1 and WAVE2 in dorsal and peripheral ruffle formation for fibroblast cell migration. Dev Cell 5: 595–609. 14536061 10.1016/s1534-5807(03)00297-1

[23] Hernandez SE, Krishnaswami M, Miller AL, Koleske AJ (2004) How do Abl family kinases regulate cell shape and movement? Trends Cell Biol 14: 36–44. 14729179 10.1016/j.tcb.2003.11.003

[24] Aspenstrom P (1997) A Cdc42 target protein with homology to the non-kinase domain of FER has a potential role in regulating the actin cytoskeleton. Curr Biol 7: 479–487. 9210375 10.1016/s0960-9822(06)00219-3

[25] Angers-Loustau A, Cote JF, Charest A, Dowbenko D, Spencer S, et al (1999) Protein tyrosine phosphatase-PEST regulates focal adhesion disassembly, migration, and cytokinesis in fibroblasts. J Cell Biol 144: 1019–1031. 10085298 10.1083/jcb.144.5.1019 PMC2148201

[26] Backsch C, Rudolph B, Steinbach D, Scheungraber C, Liesenfeld M, et al (2011) An integrative functional genomic and gene expression approach revealed SORBS2 as a putative tumour suppressor gene involved in cervical carcinogenesis. Carcinogenesis 32: 1100–1106. 21602178 10.1093/carcin/bgr093

[27] Mestre-Escorihuela C, Rubio-Moscardo F, Richter JA, Siebert R, Climent J, et al (2007) Homozygous deletions localize novel tumor suppressor genes in B-cell lymphomas. Blood 109: 271–280. 16960149 10.1182/blood-2006-06-026500

[28] Stein L, Rothschild J, Luce J, Cowell JK, Thomas G, et al (2010) Copy number and gene expression alterations in radiation-induced papillary thyroid carcinoma from chernobyl pediatric patients. Thyroid 20: 475–487. 19725780 10.1089/thy.2009.0008

[29] Akamatsu M, Aota S, Suwa A, Ueda K, Amachi T, et al (1999) Vinexin forms a signaling complex with Sos and modulates epidermal growth factor-induced c-Jun N-terminal kinase/stress-activated protein kinase activities. J Biol Chem 274: 35933–35937. 10585480 10.1074/jbc.274.50.35933

[30] Zhang M, Liu J, Cheng A, Deyoung SM, Chen X, et al (2006) CAP interacts with cytoskeletal proteins and regulates adhesion-mediated ERK activation and motility. EMBO J 25: 5284–5293. 17082770 10.1038/sj.emboj.7601406 PMC1636617

[31] Tujague M, Thomsen JS, Mizuki K, Sadek CM, Gustafsson JA (2004) The focal adhesion protein vinexin alpha regulates the phosphorylation and activity of estrogen receptor alpha. J Biol Chem 279: 9255–9263. 14625289 10.1074/jbc.M312160200

[32] Kioka N, Ito T, Yamashita H, Uekawa N, Umemoto T, et al (2010) Crucial role of vinexin for keratinocyte migration in vitro and epidermal wound healing in vivo. Exp Cell Res 316: 1728–1738. 20361963 10.1016/j.yexcr.2010.03.019

[33] Ribon V, Herrera R, Kay BK, Saltiel AR (1998) A role for CAP, a novel, multifunctional Src homology 3 domain-containing protein in formation of actin stress fibers and focal adhesions. J Biol Chem 273: 4073–4080. 9461600 10.1074/jbc.273.7.4073

[34] Fernow I, Tomasovic A, Siehoff-Icking A, Tikkanen R (2009) Cbl-associated protein is tyrosine phosphorylated by c-Abl and c-Src kinases. BMC Cell Biol 10: 80. 19891780 10.1186/1471-2121-10-80 PMC2777869

[35] Mitsushima M, Takahashi H, Shishido T, Ueda K, Kioka N (2006) Abl kinase interacts with and phosphorylates vinexin. FEBS Lett 580: 4288–4295. 16831423 10.1016/j.febslet.2006.06.072

[36] Ridley AJ, Schwartz MA, Burridge K, Firtel RA, Ginsberg MH, et al (2003) Cell migration: integrating signals from front to back. Science 302: 1704–1709. 14657486 10.1126/science.1092053

