# Supplementary figures and images for: Oligomerization and Phosphorylation Dependent Regulation of ArgBP2 Adaptive Capabilities and Associated Functions
Source: PLoS One. 2014 Jan 27;9(1):e87130. doi: 10.1371/journal.pone.0087130 (PMC3903627; doi:10.1371/journal.pone.0087130)

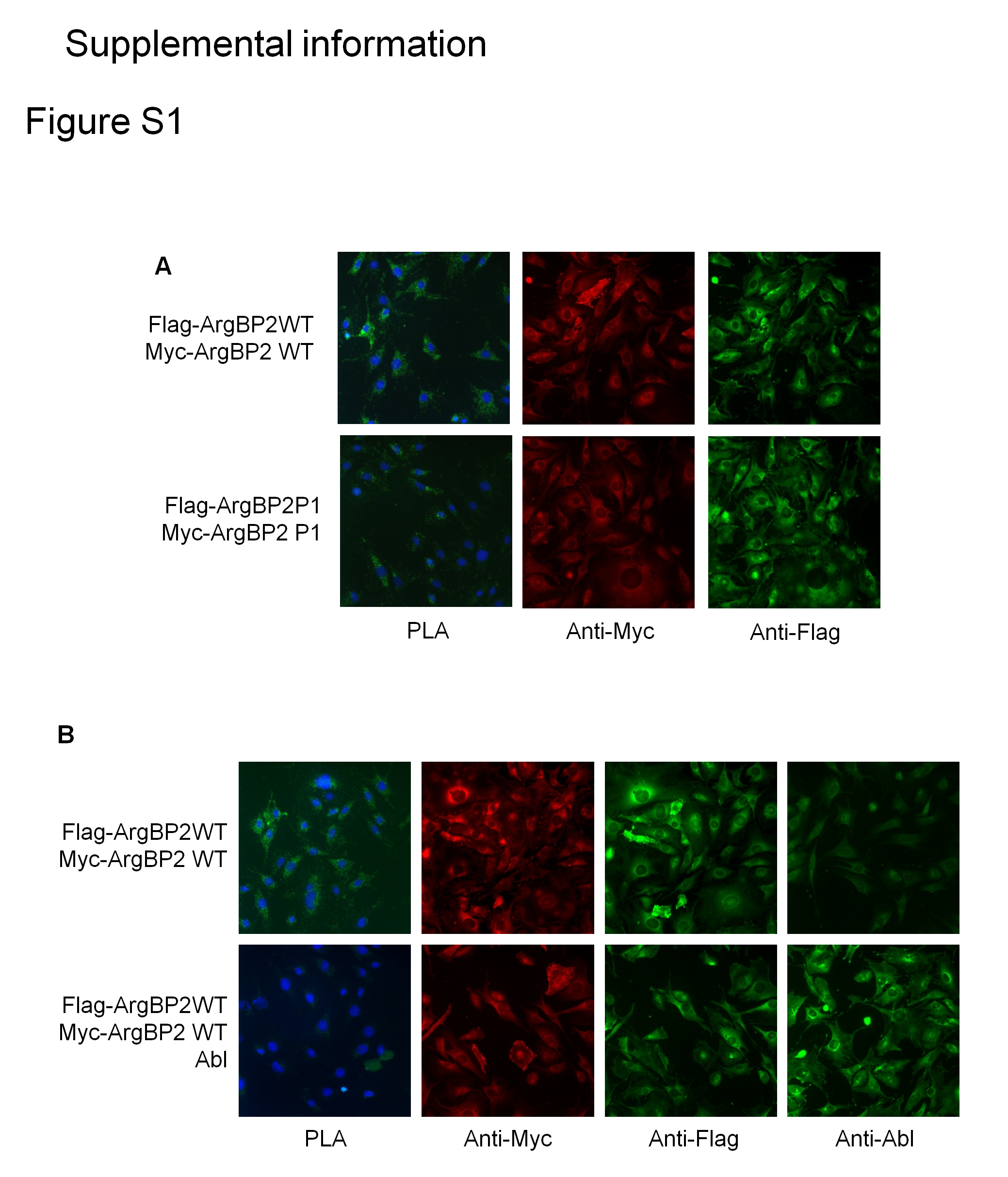

Supplement: Figure S1 — Control immunofluorescence of NIH3T3 cells used for PLA assays. (A) Control immunofluorescence corresponding to Figure 3C. Cells were stained with rabbit anti-Flag and mouse anti-Myc antibodies, followed by staining with the corresponding fluorescent secondary antibodies (Alexa-546 and 488). (B) Control immunofluorescence corresponding to Figure 5C. Cells were stained with rabbit anti-Flag and mouse anti-Myc antibodies, or with anti-Abl antibodies, followed by staining with the corresponding fluorescent secondary antibodies (Alexa-546 and 488). (TIF) [file pone.0087130.s001.tif]
